# Laboratory Changes Supporting a Diagnosis of Immune‐Mediated Anemia in Cats With Initial Suspicion of Progressive Feline Leukemia Virus

**DOI:** 10.1111/vcp.70137

**Published:** 2026-08-09

**Authors:** Nicholas Kelly, Katie McCallum, Will Bayton, Tyler Willard, Cassia Hare

**Affiliations:** ^1^ Department of Veterinary Medicine University of Cambridge Cambridge UK; ^2^ Dick White Referrals Six Mile Bottom UK

## Abstract

**Background:**

Progressive feline leukemia virus (FeLV) infection and anemia of suspected immune‐mediated origin (ASIMO) are associated with similar hematologic abnormalities, including anemia, neutropenia, and lymphocytosis. Cats with ASIMO may test positive for FeLV using point‐of‐care (POC) ELISA tests.

**Objective:**

To describe laboratory changes that may result in the misclassification of cats with ASIMO as having progressive FeLV.

**Methods:**

Cats with ASIMO and laboratory results mimicking FeLV infection were retrospectively identified via searches of laboratory and clinical records. Inclusion criteria were: 1) a positive POC FeLV result with negative confirmatory testing, 2) hematocrit < 20%, with or without macrocytosis, neutropenia, lymphocytosis, or suspicion of circulating atypical cells, and 3) a clinical diagnosis of ASIMO. Clinical and laboratory data, treatment, and outcome were recorded.

**Results:**

Nine cats met the inclusion criteria. All were vaccinated against FeLV. Seven (78%) had outdoor access and two (22%) were indoor only. Hematocrit at presentation ranged from 7‐16%, and macrocytosis was recorded in seven cases (78%), of which six were normochromic by analyzer data. Seven cats (78%) had one or more laboratory indicators of hemolysis, including an abnormal RBC histogram in all five cases in which this was available for review. Other hematologic abnormalities included neutropenia (*n* = 6, 67%), lymphocytosis (*n* = 1, 11%), and circulating atypical cells (*n* = 1, 11%).

**Conclusions:**

Cats with ASIMO may present with macrocytic, normochromic anemia and other laboratory features mimicking progressive FeLV. Evaluation of RBC histograms alongside detailed blood film evaluation is helpful in identifying immune‐mediated anemia as a likely diagnosis.

## 1 Introduction

Feline Leukemia Virus (FeLV) is known for its association with lymphoproliferative neoplasia and myelosuppression [1, 2]. Initial diagnostics rely upon detection of viral p27 antigen in blood, often using point of care (POC) ELISA tests [3], with confirmatory testing such as PCR advised on receipt of a positive result [4]. Progressive FeLV infection, whereby cats are persistently viremic [2], is associated with hematologic sequelae including neutropenia, lymphocytosis or lymphopenia, macrocytic anemia and thrombocytopenia [2, 5, 6]; this stage of infection carries a grave prognosis, potentially resulting in euthanasia [7, 8]. Similar hematologic changes, as well as positive POC FeLV results [9, 10, 11], are reported in cats with non‐associative immune‐mediated hemolytic anemia (IMHA) [9, 12], and precursor‐targeted immune‐mediated anemia (PIMA) [10, 13], previously collectively termed anemia of suspected immune‐mediated origin (ASIMO) [10]. While macrocytosis due to FeLV infection results from myelodysplasia [8] and is reported in up to 53% of FeLV cases [14], it may occur due to artifact, including agglutination in immune‐mediated anemia. Despite potentially similar clinical presentations, progressive FeLV and ASIMO require different management and carry substantially different prognoses; therefore, additional laboratory methods differentiating these conditions are desirable.

Support for immune‐mediated components to feline anemia includes demonstration of a positive saline agglutination test (SAT) or direct antiglobulin test (DAT; Coombs test), evidence of hemolysis including hyperbilirubinemia with absence of other causes, hemoglobinemia, hemoglobinuria or erythrocyte ghosts, the latter identified on blood film evaluation [15] but potentially overlooked depending on their numbers and experience of observers. Data displayed in histograms or scatter plots obtained from automated hematology analyzers may provide further evidence of immune‐mediated processes, specifically by identifying agglutination that may not be evident on blood film evaluation. These data vary between analyzers dependent on methodologies employed (i.e., impedance, flow cytometry, or both) but may include discrepancies between RBC concentration obtained via impedance and optical methods, an irregular shoulder of the RBC impedance histogram, or presence of abnormalities including a double population in the RBC histogram or cytogram, suggesting presence of agglutinates.

Anecdotally, the authors have observed several cats with final clinical diagnoses of ASIMO whereby the clinical presentation mimicked progressive FeLV infection, causing an initial diagnostic dilemma. Following review of these cases, we present a series of cats with moderate to severe anemia, initially suspected to have progressive FeLV infections based on hematology and POC ELISA results but, through review of hematologic data and further investigations, ultimately diagnosed with ASIMO. We describe hematological changes, with particular emphasis on the value of hematological histograms and blood film evaluation to aid in differentiating these two disease processes.

## 2 Materials and Methods

The laboratory databases of two small‐animal referral hospitals were searched for cats that tested positive for FeLV by POC ELISA between 2017 and 2025, facilitating the identification of cats with initial laboratory data that mimicked progressive FeLV infection. Point‐of‐care kits used included FASTest FeLV‐FIV (Vetlab Supplies Ltd., Pulborough, UK) and SNAP FIV/FeLV Combo (IDEXX Laboratories, Westbrook, Maine, USA). Inclusion required subsequent negative confirmatory FeLV testing by PCR for proviral DNA (The Veterinary Pathology Group, Exeter, UK).

Cats were considered to have hematologic presentations compatible with progressive FeLV if they had moderate or severe anemia (HCT < 20%) with or without macrocytosis, neutropenia, lymphocytosis, and/or identification of circulating atypical cells.

Laboratory testing (including bone marrow cytology and histopathology, where performed) was undertaken in on‐site laboratories of the referral institutions, overseen by board‐certified veterinary clinical pathologists. Hematology analysis was performed using either Sysmex XN‐1000 V (Sysmex Corporation, Kobe, Japan) or Siemens ADVIA 2120 (Siemens, Munich, Germany) analyzers, with initial blood film evaluation undertaken by trained laboratory technicians with or without input from clinical pathologists. Results were interpreted in line with institution‐specific reference intervals. Where requested, saline agglutination testing (SAT) was performed according to laboratory standard operating procedure and direct antiglobulin test (DAT; Coombs test) was performed using a commercially available gel column test kit (Gel Test feline DAT, Alvedia, Limonest, France). Biochemical testing at both institutions utilized a Beckman AU480 analyzer (Beckman Coulter, Brea, California, USA).

Medical records were reviewed under the direction of a board‐certified specialist in internal medicine. Details recorded included signalment, vaccination and roaming status, results of diagnostic imaging investigations, treatment and follow‐up. Cats were considered to have clinical diagnoses of ASIMO based on the presence of consistent hematologic (and where available, bone marrow) findings, exclusion of other causes of anemia, and decisions to start immunosuppressive treatment.

### 2.1 Ethical Approval

Ethics approval was obtained through the University of Cambridge, Department of Veterinary Medicine Ethics and Welfare Committee (CR675).

## 3 Results

A total of nine cats met inclusion criteria. Eight cats were Domestic Shorthairs, and one was a Balinese crossbreed. Four cases were male; five cases were female; all were neutered. Age ranged from 9 months to 9 years. Seven cats had outdoor access; two cats were kept exclusively indoors. All cats were up to date with routine vaccinations against FeLV, Feline Herpesvirus (FHV), Feline Calicivirus (FCV), and Feline Panleukopenia Virus (FPV).

Hematologic data are summarized in Table 1. Hematocrit ranged from 7% to 16% (mean 12%) at initial presentation. Macrocytosis, as evidenced by increased MCV, was recorded in seven cases (7/9; 78%). Anemias were normochromic based on MCHC in eight cases (8/9; 89%), and a total of six cats (6/9; 67%) were classified as having a macrocytic, normochromic anemia based on analyzer data. Whilst four cats had reticulocytosis and accompanying polychromasia, this was not deemed a sufficient regenerative response (based on degree of anemia) in any case. The blood film documented erythrocyte ghosts in five cases (5/9; 56%) and microscopic agglutination in one case (1/9; 11%).

**TABLE 1:** Hematology data.

Case	Analyzer	RBC (×10^12^/L) (RI 5–10)	MCV (fL) (RI 39–55)	MCHC (g/dL)(RI 30–36)	Reticulocytes (×10^9^/L) (RI 0–60)	HCT (%) (RI 26–45)	WBC (×10^9^/L) (RI 5.5–19.5)	Neutrophils (×10^9^/L) (RI 2.5–12.5)	Lymphocytes (×10^9^/L) (RI 1.5–7)	Platelets (×10^9^/L) (RI 200–800)	SAT	DAT	Additional hematological features supportive of agglutination/hemolysis
1	Sysmex	2.3	68	31.2	13	16	7.2	2.1	4.6	77	Negative	Negative	Erythrocyte ghosts Irregular RBC histogram
2	Sysmex	2.4	64.5	30.1	122	16	29.1	23.7	2.3	161	Negative	Negative	Irregular RBC histogram
3	Sysmex	2.0	65.5	32.1	44	12	10.6	4.8	4.1	146	NP	Positive	Irregular RBC histogram
4	Sysmex	2.2	66.5	33.6	10	14	16.1	1.9	13.1	49	NP	NP	Erythrocyte ghosts Microscopic agglutination Irregular RBC histogram
5	Sysmex	1.7	61.1	24.5	115	10	23.1	16.9	3.7	270	NP	Positive	Erythrocyte ghosts Irregular RBC histogram
6	ADVIA	3.2	51.3	32.3	40	16	9.9	2.0	6.8	79	Negative	NP	NR
7	ADVIA	1.3	57.3	30.6	68	8	2.6	0.6	1.8	40	NP	NP	Erythrocyte ghosts
8	ADVIA	2.2	49.4	30.8	63	11	6.2	2.4	3.5	NR due to marked clumping	NP	NP	Erythrocyte ghosts
9	ADVIA	1.0	73	31.3	34	7	5.1	1.2	3.7	NR due to marked clumping	NP	NP	NR
Mean		2.0	61.8	30.7	57	12	12.2	6.2	4.8	117			

*Note:* For simplicity, reference intervals provided are for the laboratory using the Sysmex XN‐V analyzer; however, all abnormalities were outside the reference intervals of both laboratories. Values above the reference intervals (or positive on SAT/DAT) are highlighted in a red box; values below the reference intervals are highlighted in a blue box.

Abbreviations: NP, not performed; NR, not reported; RI, reference interval.

Retrospective review of RBC histograms from cases processed with the Sysmex XN‐V documented abnormalities suggestive of agglutination in 5/5 cases (100%), despite this not being identified on blood film evaluation in four of these. An irregularly spiked RBC histogram peak was evident in all cases (Figure 1B), and in one case, the RBC histogram additionally had a flattened appearance, giving the appearance of an uneven plateau (Figure 1C). This was interpreted as providing support for an immune‐mediated component to the anemia. There was no record of scattergram abnormalities for cases processed using the ADVIA 2120, and data were not available for retrospective review.

**FIGURE 1:**
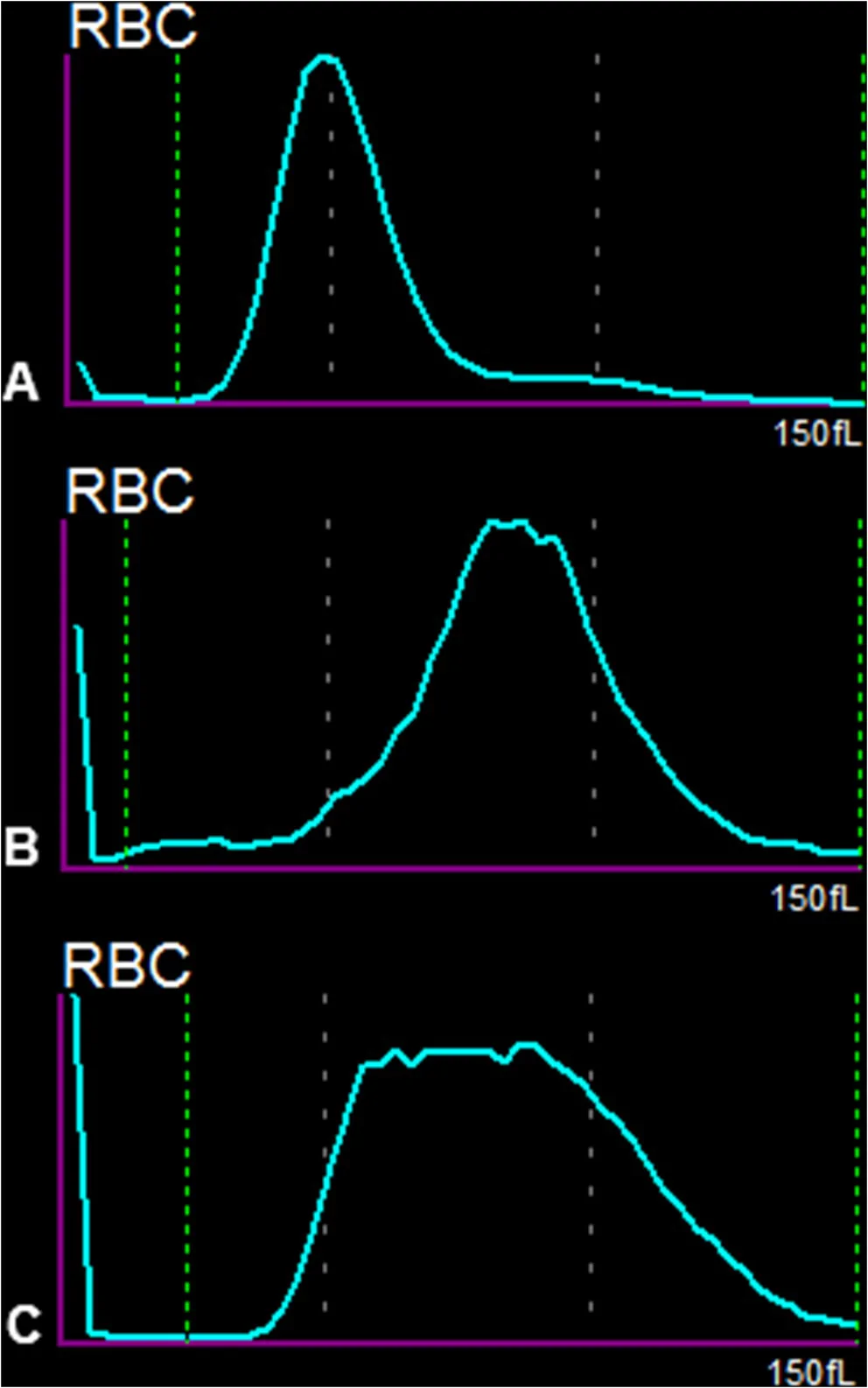
Sysmex XN‐V RBC histograms. (A) Histogram from a normal cat. (B) Histogram from Case 1, demonstrating an irregular broad, spiked peak. (C) Histogram from Case 4, demonstrating an irregular, spiked peak with a plateau appearance.

Neutropenia was present in six cases (6/9; 67%), with neutrophilia in two cases (2/9; 22%). Lymphocytosis was noted in one case (1/9; 11%) and comprised predominantly morphologically unremarkable small lymphocytes on blood film evaluation. Large, atypical cells were noted upon blood film analysis in one case (1/9; 11%), without definitive assertion between reactive or neoplastic cells. There was thrombocytopenia based on automated counts in eight cats (8/9; 89%), though two cases were subject to marked platelet clumping, so no numerical value was provided by the reporting laboratory per their standard operating procedure.

Selected biochemistry data are presented in Table 2. Common abnormalities included hyperproteinemia due to hyperglobulinemia in six cases (6/9; 67%), mild to moderate increases in ALT activity in six cases (6/9; 67%), elevated urea in five cases (5/9; 56%), and hyperbilirubinemia in three cases (3/9; 33%), considered of pre‐hepatic origin due to hemolysis in all cases.

**TABLE 2:** Biochemical data.

Case	Total Protein (g/L) (RI 56–78)	Albumin (g/L) (RI 25–43)	Globulin (g/L) (RI 24–47)	Urea (mmol/L) (RI 5.4–10.7)	Creatinine (μmol/L) (RI 56–153)	ALT (IU/L) (RI 17–62)	ALP (IU/L) (RI 10–93)	Bilirubin (μmol/L) (RI 0–11)
1	80	30	50	11.0	107	83	24	12
2	107	31	76	26.6	224	70	28	5
3	55	25	30	8.3	60	50	15	8
4	81	28	53	9.3	159	24	20	4
5	70	33	37	11.3	113	30	9	5
6	105	28	77	6.8	83	119	9	5
7	77	32	45	7.2	80	82	9	12
8	78	24	54	13.2	96	331	9	21
9	106	35	71	11.0	80	98	24	7
Mean	84	30	55	11.6	111	99	16	9

*Note:* For simplicity, the provided reference intervals are for one laboratory; however, all abnormalities were outside the reference intervals of both laboratories. Values above the reference intervals are highlighted in a red box; values below the reference intervals are highlighted in a blue box.

FIV testing (performed as part of the combination POC ELISA) was negative in all cases. Four cases (4/9; 44%) had second POC FeLV tests performed within 1 month of the first. In three cases, the second test was negative and in one case, there was an initial repeated positive result at day 2 followed by a negative result after 8 months. No follow‐up testing was performed in cats that were euthanized during the study period.

PCR testing for FeLV proviral DNA was performed on peripheral blood (*n* = 8, 89%) and/or bone marrow (*n* = 3, 33%). No cats had other forms of FeLV testing performed.

Saline agglutination testing was performed in three cases; two of these were negative and one was initially positive but negative after washing. Direct antiglobulin testing was performed in four cases, with two testing positive.

Diagnostic imaging was performed in seven cases, consisting of abdominal ultrasound, thoracic radiography, and abdominal radiography. Frequent imaging findings included splenomegaly (*n* = 7), scant peritoneal effusion (*n* = 6), mild to moderate enlargement of the cardiac silhouette (*n* = 3), hepatomegaly (*n* = 3), hyperechoic hepatic parenchyma (*n* = 3), reduction in renal corticomedullary demarcation (*n* = 3) and mesenteric lymphadenomegaly (*n* = 3). Where cytology was performed on lymph nodes, liver, and/or spleen, these showed nonspecific changes, including reactivity and extramedullary hematopoiesis.

Bone marrow cytology was performed in six cases (6/9; 67%) and core biopsy for histopathology in two cases (2/9; 22%), one of which yielded a non‐diagnostic sample. The most frequently recorded findings were ineffective erythroid hyperplasia (6/6; 100%) and rubriphagocytosis (4/6; 67%). Maturational arrest was observed in one case and occurred at the polychromatic rubricyte stage. Mild dysplastic change within the erythroid series was observed in 3/6 cases in which cytology was performed, with abnormalities including asynchronous nuclear and cytoplasmic features (*n* = 3), binucleation of precursors (*n* = 1), and rare cells resembling megaloblasts (*n* = 1). This degree of dysplasia was interpreted as being secondary to presumptive immune‐mediated disease in all cases. A left shift of the myeloid lineage, consistent with peripheral demand, was noted in two cases.

All nine cats received immunosuppressive doses of oral prednisolone for their first presentation of ASIMO; one cat received adjunct treatment with cyclosporine. Blood transfusions were administered in four cases; three were of feline blood, and the fourth was a xenotransfusion of canine blood.

Follow‐up data were variably available. Remission, defined as achieving an HCT/PCV > 25% with the absence of clinical signs [16], was achieved in 7/9 cases (78%), with one further case showing reported clinical improvement but without hematology data available for review, giving a total reported improvement rate of 89%. Relapses occurred in 3/7 cases (43%) that achieved remission and occurred multiple times in two cases. Time to remission in the first episode ranged from 19 to 121 days, with a median time to remission of 28 days. Five cats (56%) are still alive at the time of writing, and four (44%) were euthanized (median survival time 415 days [2–2600]). Two cats were euthanized for signs directly attributed to ASIMO; the other two cats were euthanized due to pathologies not directly related to ASIMO; necropsies were offered in all cases but were declined by the owners.

## 4 Discussion

These cases highlight the potential for cats with ASIMO to clinically mimic those with progressive FeLV infection at initial presentation, with the presence of moderate to severe macrocytic, inadequately regenerative or non‐regenerative anemia, positive POC FeLV antigen results, and neutropenia. Negative PCR results for FeLV proviral DNA, hematological and/or bone marrow findings suggestive of an immune‐mediated component to the anemia, and in the majority of cases (89%), a positive response to immunosuppressive therapy support the clinical diagnosis of ASIMO in these cases, and awareness of this clinical overlap is vital to prevent potentially unnecessary euthanasia of patients that may respond favorably to treatment. A suggested diagnostic approach to cats presenting with anemia and a positive POC FeLV test is outlined in Figure 2.

**FIGURE 2:**
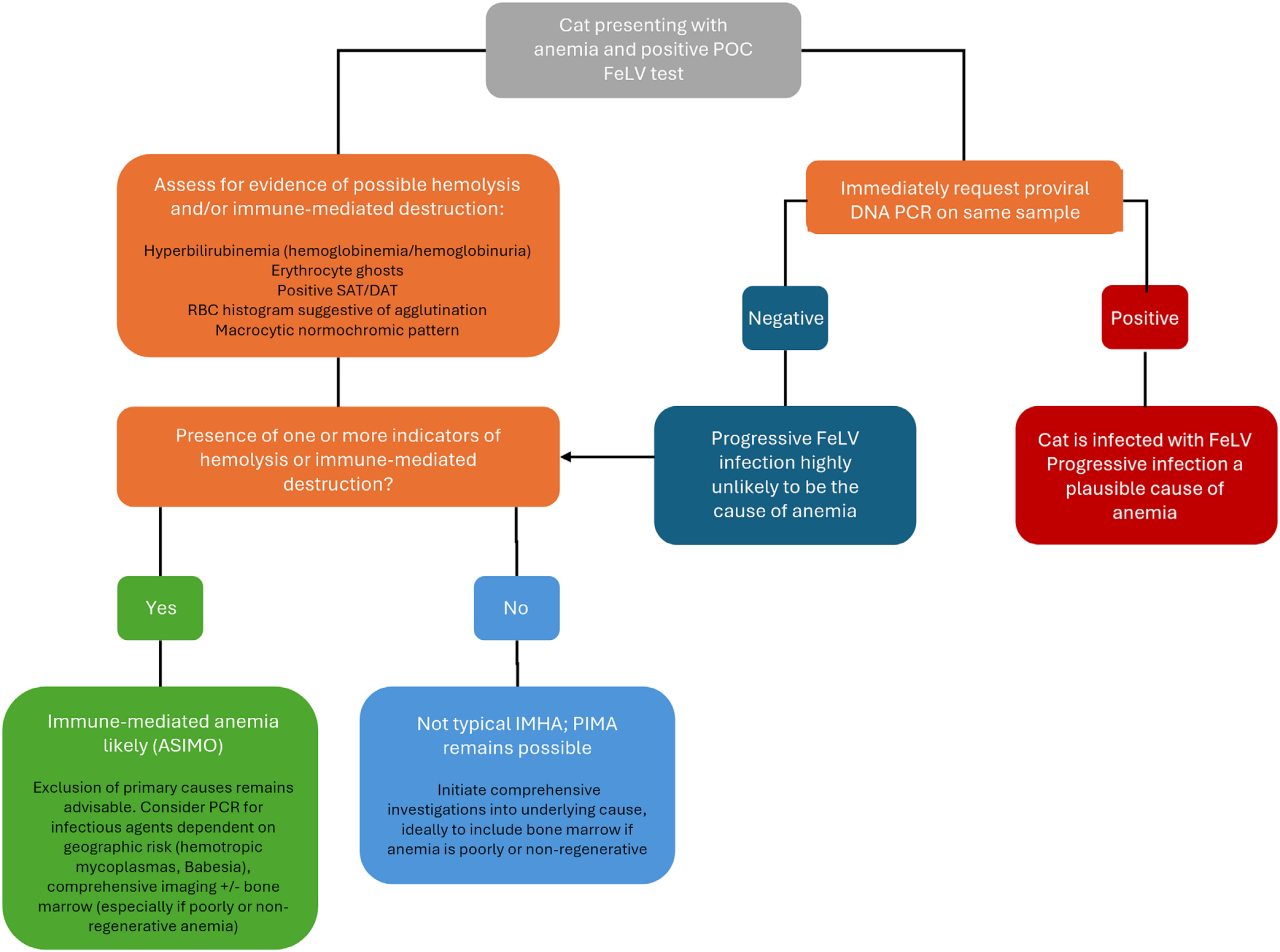
Suggested diagnostic approach to cats presenting with anemia and a positive POC FeLV result. DAT, direct antiglobulin test; SAT, saline agglutination test.

A key initial feature which drew attention to the likely immune‐mediated nature of the anemia was the abnormal appearance of the RBC histograms obtained from the Sysmex XN‐V analyzer. These histograms graph relative distributions of RBCs by size and should appear as a single smooth curve, returning to the *x*‐axis within the relevant size discriminators (Figure 1A). They are produced by analyzers using impedance technology (Coulter principle) for determination of RBC parameters. The presence of agglutinated RBCs may result in undercounting via impedance methods and a spurious increase in MCV, giving an erroneous impression of a macrocytic, normochromic anemia resembling that seen in myelodysplasia in progressive FeLV infection. Indeed, the use of suggested features such as marked macrocytosis (> 65 fL) to support diagnoses of primary myelodysplastic syndrome (MDS) [17] would have resulted in misdiagnoses of four of our cases (44%).

Whilst macrocytosis resulting from dyserythropoiesis or reticulocytosis might be expected to result in skewing of impedance RBC histograms to the right and has been reported in cats with FeLV [14], 100% of cats in our study where data was available displayed irregular jagged appearances suggestive of agglutination, which was not evident microscopically in 80% of these. Publications in the human literature describe the use of histograms, alongside other clinical and hematologic data, to increase suspicion of a variety of diseases [18, 19], and studies have shown histograms to be more sensitive indicators of macrocytosis and anisocytosis than MCV alone [20]. Whilst many veterinary clinical pathologists routinely utilize histogram and scattergram data, there is a paucity of publications regarding their clinical use in the veterinary field [14, 21, 22]. Increased understanding of these data may provide clues about likely causes of hematological abnormalities and provide additional support for an immune‐mediated process outside those described in more widely known diagnostic criteria [15].

In the context of cats with ASIMO, the potential for false positive POC FeLV results has been increasingly recognized in recent years and explanations have been postulated by other authors [9, 10, 11], with further discussion beyond the scope of this study. However, we hope to draw attention to the fact that whilst POC tests have excellent sensitivity, specificity and negative predictive value [23], their positive predictive value is typically low because of low prevalences of disease in owned cats [1, 3, 24]; data regarding prevalence should additionally be interpreted considering test methodology. Although positive predictive value is likely to have been increased by the presence of compatible clinical signs in our cases, our results further emphasize the essential need for confirmatory testing when presented with a positive result.

Our study has limitations, primarily the limited number included and the multicenter, retrospective nature of the study design. Further investigations that could have more conclusively established the FeLV status of the cats presented, such as virus isolation or sequential monitoring of retroviral status as described in the FeLV lifetime study [25], were not possible, and in several cases, further investigations, including bone marrow analysis and necropsy in cases that were euthanized, were declined by owners. Furthermore, virus isolation is infrequently used at our institutions for logistical reasons. Several authors have outlined uncertainties surrounding the pathogenesis and designation of FeLV infection as abortive, focal, regressive, and progressive [24], and we cannot definitively conclude that the cats in our cohort were not infected with FeLV. However, the positive correlation between copies of FeLV proviral DNA in whole blood and concentration of p27 antigen in plasma using quantitative PCR and ELISA tests respectively has shown PCR to be a robust confirmatory test [26, 27], and a key premise of our study is that progressive FeLV infection, the variant known to be associated with myelodysplasia and resultant hematological abnormalities, was highly unlikely in our cases considering negative PCR results. Additionally, all cats in our study were vaccinated against FeLV, and two had no outdoor access, further limiting the likelihood of infection.

In summary, we highlight potential diagnostic conundrums presented by cats with ASIMO, which, as well as having a positive FeLV POC result, may present with hematological changes highly suggestive of FeLV infection. Alongside established features such as erythrocyte ghosts, microscopic agglutination, and hyperbilirubinemia, evaluation of RBC histograms may provide additional support for an immune‐mediated process by detecting probable agglutination that may otherwise be undetected and could contribute to spurious macrocytosis. Caution is required when faced with these cases to avoid premature euthanasia of cats that may respond favorably to immunosuppressive treatment.

## Conflicts of Interest

The authors declare no conflicts of interest.

## Data Availability

The data that support the findings of this study are available on request from the corresponding author. The data are not publicly available due to privacy or ethical restrictions.
